# Evidence for Recipient-Derived Cells in Peribiliary Glands and Biliary Epithelium of the Large Donor Bile Ducts After Liver Transplantation

**DOI:** 10.3389/fcell.2020.00693

**Published:** 2020-08-05

**Authors:** Iris E. M. de Jong, Michael E. Sutton, Marius C. van den Heuvel, Annette S. H. Gouw, Robert J. Porte

**Affiliations:** ^1^Section of Hepatobiliary Surgery and Liver Transplantation, Department of Surgery, University of Groningen, University Medical Center Groningen, Groningen, Netherlands; ^2^Surgical Research Laboratory, Department of Surgery, University of Groningen, University Medical Center Groningen, Groningen, Netherlands; ^3^Department of Pathology, University of Groningen, University Medical Center Groningen, Groningen, Netherlands

**Keywords:** fluorescence *in situ* hybridization, peribiliary glands, liver transplantation, post-transplant cholangiopathy, regeneration

## Abstract

**Introduction:**

Chimerism after orthotopic liver transplantation (OLT) has largely been investigated in intrahepatic cellular constituents. However, little is known about chimerism in the extrahepatic and large intrahepatic bile ducts. Our aim was to evaluate the presence and extent of chimerism after OLT in the peribiliary glands (PBG) and the luminal epithelium of the large donor bile ducts.

**Methods:**

For this study, we examined six extrahepatic and large intrahepatic bile ducts from livers that were re-transplanted. In all cases there was a sex-mismatch between donor and recipient (female donor organ and male recipient), which allowed to discriminate between donor- and recipient-derived cells. Specimens from female to female transplants were used as negative controls and male to male transplants as positive controls. Fluorescence *in situ* hybridization (FISH) for Y and X chromosomes was performed and the percentage of XY positive cells was determined among biliary epithelial cells. Immunohistochemistry was used to correlate chimerism with histological features.

**Results:**

Cholangiocellular chimerism in all studied specimens ranged from 14 to 52%. The degree of chimerism was not associated with biliary damage. Marked chimerism was present at 5 days post-OLT. Ki-67-positivity was detected in 1–8% of the epithelial cells at the time of liver re-transplantation, and this correlated inversely with the degree of chimerism.

**Conclusion:**

Recipient-derived cholangiocytes are present in the large bile ducts of the donor liver after OLT. The presence of chimerism in the large bile ducts suggests that recipient-derived cells may play a role in biliary regeneration following ischemia-induced injury during OLT.

## Introduction

Post-transplant cholangiopathies continue to be a major challenge in clinical liver transplantation, in terms of morbidity and mortality. Incidence rates range between 1 and 30%, depending on variations in ischemia times and donor variables (Abt et al., 2003; Dubbeld et al., 2010). After cold ischemic preservation, up to 90% of the donor livers present with histological evidence of luminal epithelium injury of the extrahepatic and large intrahepatic bile ducts (op den Dries et al., 2014). Yet, a relatively small proportion of these livers develops a post-transplant cholangiopathy. Recent work suggests that this discrepancy is based on the successful versus unsuccessful regeneration of the luminal epithelium after orthotopic liver transplantation (OLT) (Karimian et al., 2013; op den Dries et al., 2014; de Jong et al., 2019).

Peribiliary glands (PBG) are progenitor/stem cell niches arranged in a three-dimensional peribiliary network in the wall of large bile ducts (Carpino et al., 2012; DiPaola et al., 2013). Besides their exocrine function, PBG cells play a pivotal role in the restoration of damaged biliary epithelium (de Jong et al., 2018; Overi et al., 2018). In an *ex vivo* model mimicking OLT, PBG cells started to proliferate 24 h after severe ischemic injury and formed new patches of biliary epithelium 72 h later (de Jong et al., 2019). This suggests that PBG contribute to epithelial healing after cold ischemic preservation-induced bile duct injury after OLT. However, in some cases also PBG cells are injured during transplantation requiring remodeling of these microscopic structures (op den Dries et al., 2014; de Jong et al., 2019).

While chimerism, characterized by the presence of recipient-derived cells among donor cells, has been shown to play a role in tissue remodeling and repair of the liver parenchyma after OLT (Theise et al., 2000b; Kleeberger et al., 2002; Hove et al., 2003), it is unknown whether chimerism also plays a role in regeneration of the biliary epithelium of the larger donor bile ducts. We aimed to assess the role of recipient-derived cells in the restoration of biliary epithelium of large donor bile ducts after OLT. In this study, we describe chimerism in both the luminal epithelium and PBG of the donor large bile ducts after OLT. We propose a thus far undescribed origin of recipient-derived cells and speculate that post-OLT remodeling of the PBG network contributes to chimerism in the large ducts. This finding may have important implications for our understanding of the pathogenesis of post-transplant cholangiopathies.

## Materials and Methods

### Preparation of Human Large Bile Duct Specimens

Tissue from the large donor bile duct (proximal to the anastomosis) of six adult male patients that previously received a female full-sized liver graft were obtained during re-transplantation. Appropriate negative controls (female recipients that received a female donor liver, *n* = 3) and positive controls (male recipients that received a male donor liver, *n* = 3) were included. The reason for re-transplantation in the study group included distinct pathological liver and bile duct conditions (Table 1). The diagnosis for post-transplant cholangiopathy was made clinically by endoscopic retrograde cholangiopancreatography (ERCP), magnetic resonance imaging (MRI) or percutaneous transhepatic cholangiography. Post-transplant cholangiopathy was defined as strictures, dilatations, or irregularities of the intra- or extrahepatic bile ducts of the liver graft in absence of hepatic artery thrombosis. The pathological conditions of the explanted liver and bile duct were confirmed histologically after re-transplantation. In our center, liver and bile duct explants are routinely biopsied and stored for analysis. Archived formalin-fixed, paraffine embedded (FFPE) tissue blocks from liver re-transplantations performed between 2007 and 2013 were retrieved. Paraffin-embedded tissue blocks were cut in specimens of 3 μm thickness and attached to adhesive glass slides. All procedures and the use of tissue specimens were performed according to recent national guidelines.

**TABLE 1:** Patient characteristics.

**Study Number**	**Diagnosis**	**Reason re-OLT**	**Recipient age**	**Warm ischemia times first OLT hrs:min**	**Cold ischemia times first OLT hrs:min**	**Days between OLT and re-OLT**	**Donor age**	**Donor type**
1	Haema- chromatosis	PTC	53	0:45	7:18	2255	45	DBD
2	HCV cirrhosis	Recurrence HCV	48	0:55	9:34	2145	41	DBD
3	PSC	PNF	66	1:30	5:27	5	74	DCD
4	Liver cirrhosis	PTC	50	0:43	8:31	1885	53	DBD
5	Liver cirrhosis	PTC	68	0:40	8:51	84	62	DCD
6	PSC	Recurrence PSC	50	0:37	8:55	4290	54	DBD

*CMV, cytomegalovirus; DBD, donation after brain death; DCD, donation after circulatory death; HCV, hepatitis C virus; OLT, orthotopic liver transplantation; PNF, primary non-function; PSC, primary sclerosing cholangitis; PTC, post-transplant cholangiopathy; Re-OLT, liver re-transplantation.*

### Immunohistochemistry and Histo-Morphology

Sections were prepared for hematoxylin and eosin (H&E) staining for histological injury assessment. All bile duct sections were examined in a blinded fashion by two experienced liver pathologists (MVDH and ASHG). Immunohistochemistry for cytokeratin 19 (CK19), von Willebrand factor (vWF), alpha-smooth muscle actin (α-SMA), Ki-67 and cluster of differentiation 45 (CD45) was performed for histological characterization of the sections. The immunostaining for CD45 was performed by using an automated immunostaining system (Benchmark ULTRA; Roche Ventana Medical Systems, Tucson, AZ, United States) and by applying the Ultraview DAB detection kit of the same company. For all other immunostainings, tissue sections were deparaffinized through a graded alcohol series and rinsed in phosphate-buffered saline (PBS, pH 7.4). Endogenous peroxidase activity was blocked by a 30-min incubation in H_2_O_2_. Antigen retrieval for CK19 and α-SMA was performed with Tris–HCl pH 9.0 buffer in a stove (80°C) overnight, for vWF with 0.2% pepsine pH 2.0 and for Ki-67 with Tris–EDTA pH 9.0 buffer in the microwave for 15 min. After PBS for 5 min, antibodies for CK19 (CK19; Abcam, Cambridge, United Kingdom, dilution of 1:100), vWF (vWF; DAKO, Glostrup, Denmark, dilution of 1:250), α-SMA (α-SMA, Sigma-Aldrich, Steinheim, Germany, dilution 1:10.000), and Ki-67 (Ki-67; DAKO, Glostrup, Denmark, dilution of 1:300) were applied for an hour. Next, a 30 min-incubation was applied with peroxidase-labeled goat anti-rabbit antibody (for CK19 and vWF) or rabbit anti-mouse antibody (for α-SMA and Ki-67) in dilution 1:100. Rabbit anti-goat for CK19 and vWF and goat-anti rabbit for α-SMA and Ki-67 were used as third antibodies (1:100 dilution). The staining reaction was developed by 3,3’-diaminobenzidine (DAB) and counterstained with hematoxylin. Stained slides were scanned by a digital scanner and processed by ImageScope. The relative number of destroyed PBG was estimated by two (blinded) independent researchers and a semi-quantitative (SQ) score was applied (0 ≤ 1%; 1 = 1–25%; 2 = 25–50%; 3 = 50–75%; 4 = 75–100%). Likewise, a SQ score for the number of inflammatory cells around PBG in the section was evaluated by two independent researchers, but a different SQ score was appropriate for the observations (the cross-section with the least inflammatory cells around PBG scored 1 and the section with the most inflammatory cells around PBG scored 5, the other sections were compared with these two extremities). To quantify vWF, α-SMA, and Ki-67 expression, we used QuPath v0.2.0 to develop an appropriate classifier for each staining (Bankhead et al., 2017). For vWF and α-SMA we determined the positive pixel count with respect to all counted pixels and for Ki-67 the positive cell count was calculated with respect to all counted cells (i.e., the proliferation index). Thereafter, a score 1–3 was applied to the outcomes to show relative expression of the markers.

### Fluorescence *in situ* Hybridization for X and Y Chromosomes

Tissue sections were deparaffinized in xylene for 2×15 min, dehydrated through a graded alcohol series, and then rinsed with distilled water for 4 min. We used a tissue digestion kit for all pretreatment and washing steps (product number: KBI-60004, Kreatech Biotechnology, Leica Microsystems, Amsterdam, Netherlands). Sections were incubated at room temperature for 30 min with 0.2 HCl and rinsed with distilled water. Then, to make DNA accessible for hybridization, tissue slides were treated with 8% sodium thiocyanate at 84 degrees for 40 min, rinsed with 2xSSC, and incubated at room temperature with a pepsin solution for 30 min that was 3 times renewed. Next, slides were rinsed with distilled water and 2xSSC followed by dehydration and air drying. 7–10 μl of a mixture of X [DXZ1, conjugated with Platinum-*Bright*495 (green)] and Y [DYZ3, conjugated with Platinum-*Bright*550 (red)] probes (Leica Microsystems, Amsterdam, Netherlands) was applied to the air-dried sections. A cover slip was applied and sealed with Fixogum. Denaturation and hybridization were performed in a humidified chamber (Thermobrite, Leica Biosystems, Amsterdam, Netherlands) at 80 degrees for 5 min and at 37 degrees for 16 h, respectively. Next, sections were covered in 2xSSC/0.1% Igepal at room temperature for 2 min followed by incubation with 0.4xSSC/0.3% Igepal at 72 degrees for 2 min and incubation with fresh 2xSSC/0.1% Igepal at room temperature for 2 min. Sections were dehydrated, airdried and counterstained with 4,6-diamidino-2-phenylindole (DAPI)/antifade 1 μg/ml.

### Fluorescence *in situ* Hybridization Analysis

Fluorescence microscopy was performed using an Olympus BX63 microscope (Olympus corporation, Leiderdorp, Netherlands) equipped with a CCD video camera and Bioview duet-3 Software. We first scanned the cross-section at low power to allow for a wide field of view. To confirm that the counted cells were classified as epithelial cells – and not inflammatory cells - we performed immunohistochemistry for CK19 and CD45 prior to fluorescence *in situ* hybridization (FISH) on the same section. The sections stained with CK19 and CD45 were first scanned with the Bioview duet-3 Software, put in xylene for 1 week, and, then, the sections were treated with FISH and scanned again with the same software. Localization of PBG and luminal epithelium was confirmed by the matching procedure in which the fluorescence section was matched with histology. A total of 25 fields of interest were selected using both the histology and the fluorescence scan. Next, 25 photomicrographs of each section were taken with green and red filter sets of Vysis (Abbott Molecular, Illinois, IL, United States). All photomicrographs were checked for accuracy. Photomicrographs in which no PBG and luminal epithelium were present or not well-recognizable were excluded. In addition, photomicrographs which showed areas without red and green signal were also excluded. In the end, two to nine photomicrographs of each biopsy in the study group were analyzed. Nuclei in which a red signal (Y chromosome) and green signal (X chromosome) were present were counted as recipient-derived cells. Nuclei that contained two green signals (X chromosomes) were counted as native donor cells. Nuclei that did not show clear signals were excluded from the counting.

### Statistical Analysis

Continuous variables were presented as mean ± standard deviation. GraphPad Prism 8 (GraphPad Software, La Jolla, CA, United States) was used for presenting data in graphs. The Spearman correlation test and the Mann-Whitney U test to calculate significance for non-parametric data were performed using SPSS software version 23 for Windows (SPSS, Inc., Chicago, IL, United States).

## Results

The initial indication for OLT, reason for re-transplantation, recipient age, ischemia times, time between primary OLT and re-transplantation, and donor age and type of the sex-mismatched transplantations are summarized in Table 1. Three patients were re-transplanted for post-transplant cholangiopathy. Recurrence of primary sclerosing cholangitis (PSC) was diagnosed in one patient and the other two patients underwent a liver re-transplantation because of primary non-function and recurrence of hepatitis C. The mean cold ischemia time for all donor livers was 8:06 ± 1:30 (hr:min) and the mean of warm ischemia times was 0:52 ± 0:20 (hr:min). The mean graft survival (time between primary OLT and re-transplantation) was 1777 ± 1594 days.

### Histological Characteristics of Large Donor Bile Ducts at the Time of Re-transplantation

**FIGURE 1:**
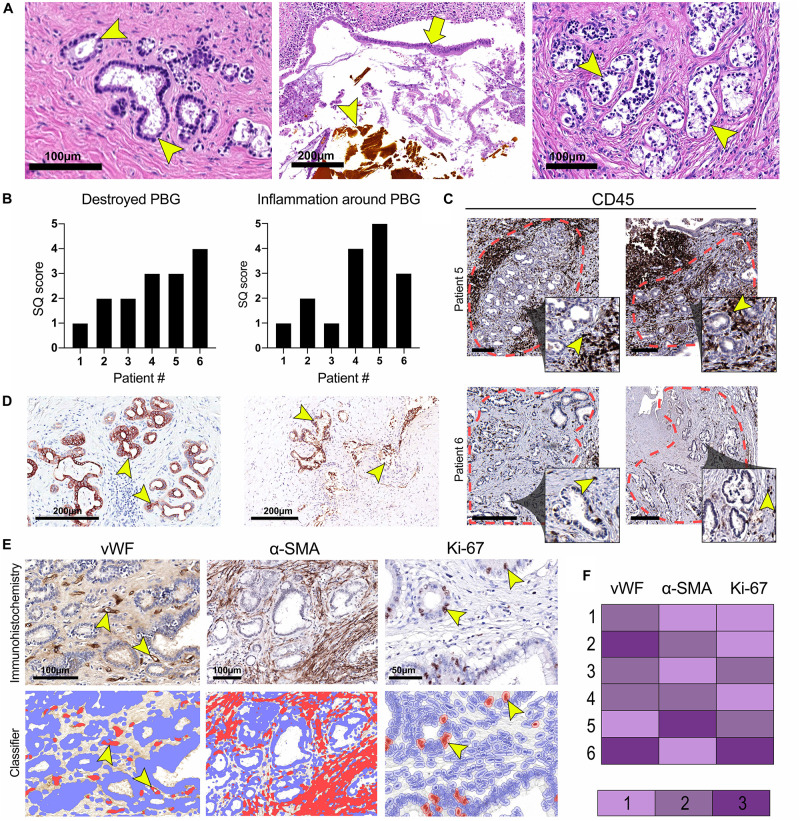
Histological characterization of large donor bile ducts from six patients that received a sex-mismatched liver graft. **(A)** Hematoxylin and eosin (H&E) staining of the large donor bile ducts; the panels from left to right correspond to numbers 2, 4, and 6 in the tables. Patient #2 required a liver re-transplantation because of recurrence of hepatitis C. Relatively intact peribiliary glands (PBG) are shown in the left panel (arrowhead), some with debris in their lumen and detached epithelial cells. Patient #4 presented with a post-transplant cholangiopathy; disrupted luminal epithelium (arrow), infiltrative inflammatory cells, and a bile cast (arrowhead) were observed (central panel). Patient #6 was re-transplanted because of recurrence of primary sclerosing cholangitis (PSC). Arrowheads point toward damaged PBG (right panel). **(B)** Semiquantitative (SQ) score of destroyed PBG and inflammation around PBG. Patients #4, #5, and #6 presented with the highest number of destroyed PBG and inflammation around PBG. **(C)** Immunohistochemistry for cluster of differentiation 45 (CD45). The red dotted line encircles clusters of PBG. Inflammation was examined within this area. The inset is depicted from the larger image and shows PBG encircled by CD45+ inflammatory cells (arrowheads). Scale bars: 250 μm. **(D)** Immunohistochemistry for cytokeratin 19 (CK19), confirming the biliary origin of the PBG cells. Arrowheads point toward CK19 positive cells. **(E)** Immunohistochemistry for von Willebrand factor (vWF), alpha-smooth muscle actin (α-SMA), and Ki-67. For all three stainings, a specified classifier was developed to determine the expression of vWF, α-SMA, and Ki-67 indicating the number of micro-vessels around PBG (i.e., microvascular density), myofibroblast activation, and proliferating cells (i.e., proliferation index), respectively **(F)** Relative expression of vWF, α-SMA, and Ki-67. Microvascular density appeared the highest in patient #2 and #6. Myofibroblast activation was most pronounced in patient #5. Patient #6 showed the highest proliferation index of the epithelial cell compartment.

The diameter of the lumens of all examined donor bile ducts ranged from 1.7 to 7.8 mm, corresponding to the common bile duct or to the right or left hepatic bile duct; collectively called large ducts. Histologically, the bile duct biopsy of patient #1, re-transplanted for a post-transplant cholangiopathy, was marked by a largely intact epithelium and mild chronic inflammation in the bile duct wall with infiltration of lymphocytes and plasma cells. PBG showed hyperplasia of both deep and periluminal PBG, mild chronic inflammation with interstitial fibrosis between the acini, and dilated ducts. The biopsy taken from the large donor bile duct in patient #2, re-transplanted for recurrence of hepatitis C, was characterized by an absent luminal epithelium. The wall showed moderate chronic inflammation with fibrotic features. The periluminal PBG were relatively intact with desquamation of the epithelium and deep PBG were absent (Figure 1A: top panel, left). Patient #3 underwent re-transplantation 5 days after the primary OLT because of primary non-function of the graft. The bile duct biopsy was characterized by a largely detached luminal epithelium but otherwise normal cytology. The bile duct wall appeared fibrotic without apparent inflammation. PBG were scarcely present; one possible remnant of periluminal PBG showed one duct and one atrophic acinus. Two deeper located PBG showed similar changes. In other areas, more PBG were present that contained interstitial fibrosis and mild inflammation. Patient #4 was re-transplanted because of a post-transplant cholangiopathy. The biliary lumen was dilated, contained a bile cast and necrotic debris (Figure 1A: top panel, center). The luminal epithelium was largely absent due to necrosis, however, the epithelial cells present were detached and showed infiltration by inflammatory cells and some nuclear atypia. The deep and periluminal PBG were surrounded and infiltrated by lymphoplasmacytic inflammatory cells. Especially the periluminal PBG were destroyed. Patient #5 underwent re-transplantation because of a post-transplant cholangiopathy. The large donor bile duct was necrotic with necrotic/bilious debris in the lumen. The luminal epithelium was largely absent and when present, partly detached without atypia. Massive inflammation was observed in the wall with lymphocytes, plasma cells, neutrophils, and macrophages. PBG were destroyed by inflammation and some remnants of ducts were seen with loss of surrounding acini. Patient #6 required re-transplantation because of recurrence of PSC. The luminal biliary epithelium was absent, and the bile duct wall was marked by mild chronic inflammation. Destruction of the periluminal PBG was observed with remnants of ducts. Deeper PBG showed interstitial fibrosis, mild to moderate inflammation, and acinar atrophy (Figure 1A: top panel, right). Hence, all biopsies showed histological damage to the biliary epithelium and wall. The biopsies corresponding to patients #4 and #5 that were re-transplanted for a post-transplant cholangiopathy showed the most extensive damage including necrosis of the biliary wall and epithelium and the presence of bile casts. Damage to PBG ranged from almost none (Figure 1A: top panel, left) to severe (Figure 1A, top panel, right). Quantification of damaged PBG showed that the highest number of destroyed PBG were found in biopsies from patients #4, #5, and #6. In the same patients, inflammation around PBG appeared more extensive, compared to the other patients (Figures 1B,C). PBG cells in bile ducts of all patients expressed CK19 which confirms the biliary origin of these cells (Figure 1D). Next, we evaluated the presence of micro-vessels around PBG (i.e., microvascular density), activation of myofibroblasts around PBG, and the proliferation index in both the PBG and the luminal epithelium using an appropriate classifier for each staining (Figures 1E,F). The endothelial cell marker vWF showed the highest microvascular density in biopsies of patients #2 and #6 (5.2 ± 3.3% positive pixels). In addition, myofibroblasts were massively activated around PBG in patient #5 (31.0 ± 17.6% positive pixels) and the highest percentage of proliferating cells was found in the biopsy of patient #6 (2.7 ± 2.7% positive cells) (Figure 1F).

### Recipient-Derived Cells Are Present in the Large Donor Bile Ducts

**FIGURE 2:**
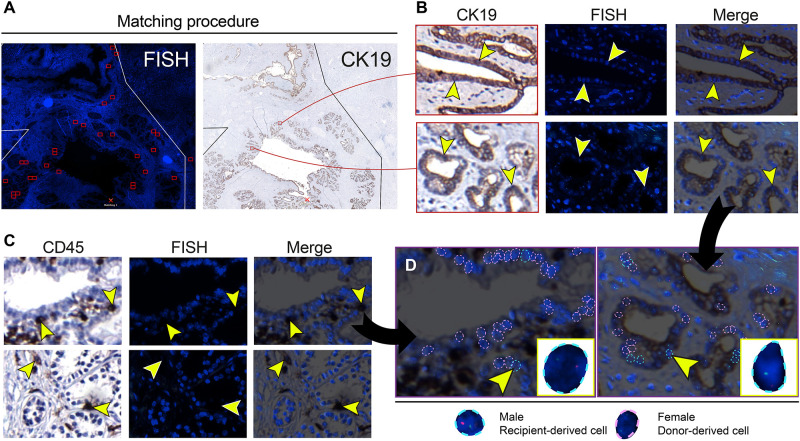
Cells that were included in the chimerism counting of the large donor bile ducts showed biliary commitment. **(A)** Matching procedure to match the fluorescence *in situ* hybridization (FISH)-treated sections with immunohistochemistry. We used Bioview Duet-3 software to match an image of the FISH-treated sections with immunohistochemistry allowing us to locate high-power FISH photomicrographs within the section and to determine cytokeratin 19 (CK19) expression of individual FISH-treated cells. Two red-colored rectangles from the FISH overview are shown in the CK19 overview in the right image. **(B)** CK19 immunostainings and FISH photomicrographs of PBG; the two CK19 immunostainings in the left images correspond with the two red-colored rectangles in the CK19 overview in panel A. CK19 is a marker for biliary epithelial cells. Images that are horizontal aligned show the same area in the cross-section. Arrowheads in the left image point toward CK19+ cells, these same cells are pointed out in the FISH photomicrograph and the merged image on the right side. These CK19+ epithelial cells were included in the chimerism counting. **(C)** Immunohistochemistry for cluster of differentiation 45 (CD45) with matching FISH photomicrographs. CD45 is a marker for inflammatory cells. Images that are horizontal aligned show the same area in the cross-section. Arrowheads point toward CD45+ cells in the CD45 immunostaining, FISH photomicrograph, and the merged image. **(D)** Chimerism counting in CD45 and CK19 immunostainings merged with FISH photomicrographs. Black arrows show that the left and right image in panel D are magnifications of the upper CD45-FISH merged image in panel C and the lower CK19-FISH merged image in panel B, respectively. Male recipient-derived cells, encircled by a blue dotted line, present with red and green spots indicating Y and X chromosomes, respectively. Female donor-derived cells, encircled by a pink dotted line, present with two green dots indicating two X chromosomes. In the image on the left side, a CD45 negative PBG cell is depicted by an arrowhead (see also corresponding images in panel **C**) and magnified in the yellow box in the lower right corner. This recipient-derived cell can therefore be classified as a non-inflammatory PBG cell (i.e., cholangiocyte). In the image on the right side, a CK19+ epithelial cell is pointed out by an arrowhead and magnified in the yellow box in the lower right corner. Cells that are encircled by a blue or pink dotted line were included in the chimerism counting. **(B–D)** Original magnification ×60.

FISH analysis of X- and Y-chromosomes was used to detect male recipient-derived cells in the female donor bile ducts. Fluorescence and histology images were matched (Figure 2A) in order to make an overlay with two exactly the same images (Figure 2B). Using this method, cells that were counted to calculate cholangiocellular chimerism were CK19+. Cells that stained positive for CD45, a marker for inflammatory cells (e.g., macrophages, neutrophils, plasma cells, and lymphocytes) were excluded from the chimerism counting. Cells that were counted as recipient-derived epithelial cells (XY) or as donor-derived epithelial cells (XX) were negative for CD45 (Figure 2C and Supplementary Figure 1). Figure 2D shows that counted cells were CD45- and CK19+, confirming that recipient-derived cells with biliary commitment were present in the large donor bile ducts. The smaller panels represent recipient-derived cells negative for CD45 (left panel) and positive for CK19 (right panel).

**FIGURE 3:**
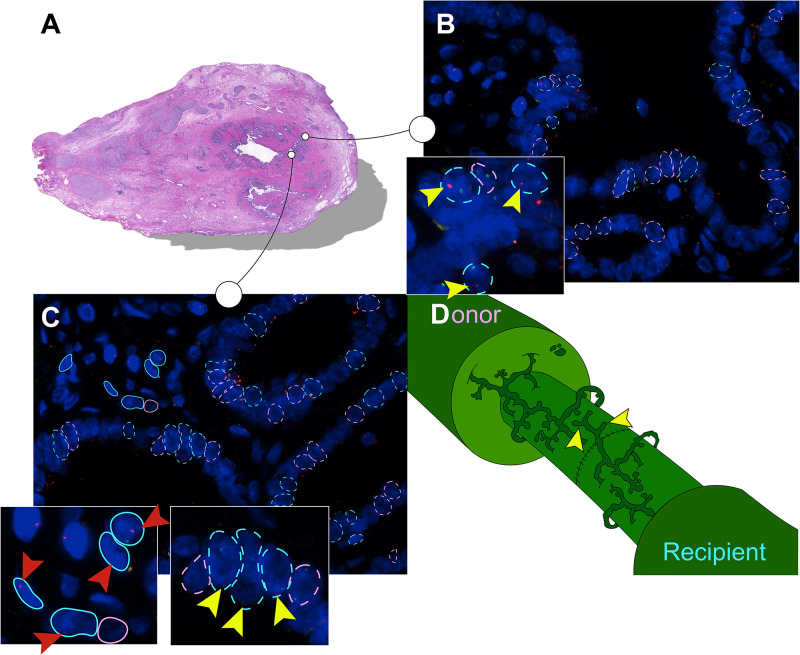
Recipient-derived cells in PBG of the large donor bile ducts. **(A)** Hematoxylin and eosin (H&E) staining corresponding with number 1 in the tables. High-power photomicrographs were obtained from different sites of the bile duct sample; two photomicrographs of this cross-section are displayed in panels **(B,C)**. The sites marked in the H&E staining correspond with the photomicrographs in panel **(B,C)**. **(B)** FISH photomicrograph. Male recipient-derived cells, encircled by a blue dotted line, present with red and green spots indicating Y and X chromosomes, respectively. Female donor-derived cells, encircled by a pink dotted line, present with two green dots indicating two X chromosomes. Yellow arrowheads point toward red spots in the cells indicating Y chromosomes, the hallmark of recipient-derived cells. Cells that are encircled by a blue or pink dotted line were included in the chimerism counting. **(C)** FISH photomicrograph. Pink and blue dotted lines indicate epithelial cells that were included in the chimerism counting. Surrounding cells are marked with an unbroken line, these cells were not included in the chimerism counting. Yellow arrowheads point toward the red dots of PBG cells indicating Y chromosomes. Red arrowheads point toward red dots of surrounding cells indicating Y chromosomes. In both cell populations (i.e., epithelial cells and surrounding cells) multiple recipient-derived cells were observed. **(D)** Schematic drawing of the bile duct anastomosis after orthotopic liver transplantation (OLT). Microscopic structures in the large bile ducts of the donor and recipient are juxtaposed after an end-to-end anastomosis by surgical sutures. Both the recipient and the donor PBG network possess a facultative stem/progenitor cell population that is activated after loss of biliary tissue. We propose a thus far undescribed mechanism in which post-OLT remodeling of the PBG network of the recipient contributes to regeneration of the donor large bile duct (yellow arrowheads). By this means, PBG remodeling could contribute to chimerism in the luminal epithelium and PBG of the large donor bile duct. **(B,C)** Original magnification ×60.

Detected by the presence of XY chromosomes, recipient-derived cells were found in different ratios in all six large duct biopsies of patients that received a sex-mismatched liver graft (Table 2). Y-positive cells were consistently observed in the male positive controls whereas none were found in the negative controls (Supplementary Figure 2). Epithelial chimerism ranged between 14 and 52% and was present as soon as 5 days after OLT. There were no significant differences in chimerism between patients re-transplanted for biliary or non-biliary diseases. The donor livers that were explanted because of a post-transplant cholangiopathy showed relatively high percentages recipient-derived cholangiocytes (34, 43, and 25%), although the highest percentage cholangiocellular chimerism (52%) was observed in the large bile duct of a liver explanted for a non-biliary disease (Table 2). There were no significant differences in the number of recipient-derived cells counted in the luminal epithelium and PBG cells. Interestingly, some areas displayed no recipient-derived cells at all, whereas other regions of the same cross-section showed up to 30% cholangiocellular chimerism suggesting that recipient-derived cells were clonally distributed throughout the donor bile duct. None of the histological or clinical features (e.g., inflammation, destroyed PBG, microvascular density, myofibroblast activation, recipient age, ischemia times and days between transplantations) correlated with epithelial chimerism, except for the proliferation index. The percentage of positive Ki-67 cells correlated inversely with epithelial chimerism (*R2* = −0.81; *P* = 0.037). Figure 3 summarizes the present study in which matched photomicrographs were taken (Panel A) and analyzed for the presence of recipient-derived cells (Panel B, C: pointed out by yellow arrowheads and encircled by a blue dotted line). In addition, surrounding non-epithelial solitary cells of which some with elongated nuclei (i.e., mesenchymal cells) were found to be a mixed population of XY and XX genotypes (Figure 3C: pointed out by red arrowheads and encircled by an unbroken line). These surrounding cells were not included in the chimerism counting.

**TABLE 2:** Chimerism in the large donor bile ducts.

	**PBG**		**Epithelium**		**Overall**	
**Study Number**	**Count**	**%**	**Count**	**%**	**Count**	**%**
1	61/176	35	10/31	32	71/207	34
2	14/27	52	−	−	14/27	52
3	20/138	14	16/118	14	36/256	14
4	−	−	9/12	43	9/12	43
5	13/46	28	16/69	23	29/115	25
6	37/277	13	4/21	16	41/298	14
Total	145/664	22	55/264	21	200/928	22

*PBG, peribiliary glands.*

## Discussion

In the present study, we demonstrated the presence of CK19 positive and CD45 negative recipient-derived cells in the donor large bile ducts. Epithelial chimerism ranged between 14 and 52% and was observed as soon as 5 days after OLT. Evidence of cell proliferation was observed in 1–8% of the biliary epithelial cells at time of liver re-transplantation, and this correlated inversely with the extent of chimerism. Clustered localization of recipient-derived cholangiocytes suggested a clonal expansion. While chimerism after OLT has largely been investigated in intrahepatic cellular constituents, this is the first study in which cholangiocellular chimerism is demonstrated in the large donor bile ducts.

We could not find a relationship between the extent of chimerism and the time interval between OLT and sampling at re-transplantation. This is in accordance with an earlier study in which a time-dependent correlation was observed for hepatic chimerism, but not for chimerism of the intrahepatic small cholangiocytes. In that study, more hepatocellular chimerism was observed in liver biopsies taken at a longer time interval after OLT, compared to biopsies taken early after the procedure, whereas such pattern was not noted for the small intrahepatic cholangiocytes (Theise et al., 2000b). In contrast to this, the presence of recipient-derived hepatocytes has been suggested to be a relatively early event (Fogt et al., 2002; Idilman et al., 2004, 2007) or not time-dependent at all (Kleeberger et al., 2002).

The extent of biliary injury did not seem to influence the engraftment of recipient-derived cells. Moreover, microvascular density, myofibroblast activation, and inflammation were not correlated with cholangiocellular chimerism. Most severe damage was observed in the bile ducts of patients #4 and #5 and the least severe biliary damage in patient #1. All these three patients suffered from post-transplant cholangiopathies, but cholangiocellular chimerism rates in these patients were moderate, compared to the others. As reflected by the radiographic presentation of post-transplant cholangiopathies, biliary lesions are often discontinuous along the biliary tree. The morphological abnormalities we observed in the present study may therefore have been influenced by “sampling error.” Moreover, damage or loss of epithelial cells may have influenced the extent of chimerism: Loss of recipient-derived epithelial cells in the donor bile duct later in the disease development due to ongoing injuries can, unfortunately, not be quantified, thus the accumulative number of recipient-derived cells remains elusive.

Chimerism of intrahepatic small cholangiocytes in a study of Theise et al. (2000b) ranged between 4 and 38%, which is somewhat lower than the chimerism of the large cholangiocytes observed in the current study. Other studies reported even a lower number of intraparenchymal recipient-derived cells with biliary differentiation (Kleeberger et al., 2002; Hove et al., 2003). The extent of hepatocellular chimerism ranged between 2 and 40% and only some studies suggested a correlation with hepatic damage (Kleeberger et al., 2002; Hove et al., 2003; Idilman et al., 2004).

Up to date, multiple studies have demonstrated recipient-derived cells in organ allografts (Bittmann et al., 2001; Quaini et al., 2002; Boersema et al., 2009). Two questions remain unanswered as results have been equivocal and various regenerative mechanisms presumably overlap: (1) What is the origin of the recipient-derived cells? And (2) why are native donor cells replaced by recipient cells?

Regarding the first question, various theories have been proposed to explain hepatic chimerism: fetal microchimerism, bone marrow-derived stem cells, cell fusion, and circulating epithelial cells. The first one refers to fetal cells that are known to persist in the maternal circulation after pregnancy which could explain non-self cells in the donor liver after OLT (Fanning et al., 2000; Baccarani et al., 2001). Two previous studies have precluded fetal microchimerism as (single) source for the observed recipient-derived hepatocytes and intrahepatic small cholangiocytes (Hove et al., 2003; Idilman et al., 2007). Bone-marrow derived stem cells are widely accepted as an origin of intraparenchymal recipient-derived cells after OLT as this is confirmed with mouse and human experiments (Petersen et al., 1999; Theise et al., 2000a, b). Cell fusion between hematopoietic stem cells and hepatocytes has been described, but appears to be very rare and its contribution to post-OLT chimerism is therefore considered trivial (Wang et al., 2003). Circulating epithelial cells are another putative source of recipient-derived hepatic cells after OLT, but this has, actually, never been investigated in this setting (Roos et al., 2019).

Based on our results, we propose an as yet undescribed mechanism for the presence of cholangiocellular chimerism in the large donor bile ducts after OLT (see Figure 3D). PBG contain a facultative progenitor compartment that is known to have the capacity to migrate and replenish biliary luminal epithelium after severe injury (DiPaola et al., 2013; Carpino et al., 2019; de Jong et al., 2019). During an OLT, the donor and recipient extrahepatic bile duct are connected through an end-to-end anastomosis. After implantation of the liver allograft, liver and bile duct regenerate the injured and lost tissue afflicted by ischemia-reperfusion injury. In this setting, recipient PBG cells might contribute to the regeneration of the donor luminal epithelium. In an *ex vivo* human bile duct slice model, PBG cells were shown to replicate, mature and migrate toward several denuded surfaces within 3 days (de Jong et al., 2019). This finding suggests that recipient PBG cells are able to migrate through stroma contributing to PBG and luminal epithelium wound healing of the large donor bile ducts (Carpino et al., 2019). In addition, in a pig model of extrahepatic bile duct replacement by a biodegradable tubular polymer scaffold, PBG appeared to be the first cholangiocellular compartment to regenerate, followed by regeneration of the luminal epithelium (Miyazawa et al., 2005; Li et al., 2012). After 6 weeks of replacement of the extrahepatic bile duct by the scaffold, PBG-like structures were appearing in the scaffold, whereas the luminal biliary epithelium reappeared at 10 weeks. After 6 months, stroma tissue was thicker, luminal epithelium flatter, and the number of PBG was equal to that of the controls (Miyazawa et al., 2005). This suggests that PBG regeneration, or “remodeling” is a primary feature of bile duct regeneration. Following these observations, contribution of recipient PBG cells to the recovery of the donor bile duct would be a relatively late event which is not in agreement with our results that show cholangiocellular chimerism already after 5 days.

We therefore propose overlapping strategies in which cholangiocellular chimerism contributes to accomplish tissue repair: After implantation of the liver graft, bone-marrow derived cells may be recruited to regenerate intraparenchymal cells after ischemia-induced liver injury. These cells travel through the blood stream and a proportion of hemopoietic stem cells may be delivered to the liver whereas another proportion may engraft in the damaged large ducts via the peribiliary vascular plexus. Replication of engrafted hemopoietic stem cells in the donor large ducts could explain the observed clonal distribution of recipient-derived cells. Next to this process, PBG remodeling at both the recipient and donor site of the large ducts may be the primary event to regenerate luminal epithelium. In this process, PBG cells from the recipient may be mobilized and contribute to regeneration of the PBG and luminal epithelium of the donor bile duct. This putative mechanism explains the higher number of recipient-derived cells in the large bile ducts compared to small intraparenchymal cholangiocytes (Theise et al., 2000b; Kleeberger et al., 2002; Hove et al., 2003).

Regarding the second question, earlier studies (including the present one) were not able to demonstrate a clear correlation between liver and bile duct injury and the extent of post-OLT hepatocellular or cholangiocellular chimerism (Kleeberger et al., 2002; Hove et al., 2003; Idilman et al., 2004). Interestingly, in the present study, chimerism and proliferation index were inversely correlated. However, due to the relative small number of patients included in this study, the results should still be confirmed in larger series. By using sex-chromosomes for the identification of recipient-derived cells in donor bile ducts, we were restricted to male patients undergoing re-transplantation of a liver from a female donor. This limited the number of patients we could include in this study.

In addition, as applies equally for our study, detection of recipient-derived cells relies on molecular experiments (Real-Time Polymerase Chain Reaction and/or FISH) to label DNA fragments in archival frozen or paraffine-embedded specimens. These experiments are particularly challenging as proper preservation of DNA fragments requires optimal conditions during sample processing. Heterogeneity in sample processing makes quantification of recipient-derived cells therefore rather controversial and previously reported numbers may have been under- or overestimated (Aini et al., 2013).

In conclusion, this study provides evidence for the presence of recipient-derived cells in the large donor bile ducts after OLT. Recipient-derived cells may contribute to regeneration of the damaged donor bile duct and distinctive origins of these cells could explain the divergent results obtained in liver chimerism studies up to now. This study provides an opportunity for a wide array of mechanistic studies, with the ultimate goal to identify methods that can be applied to stimulate biliary regeneration. These studies should focus on the source of recipient-derived cells in the large donor bile ducts and address the question what the drivers are behind biliary chimerism. To that end, lineage tracing studies could confirm the source of recipient-derived cells and expression profiles of high- and low-chimerism PBG could be compared for deeper insights regarding chimerism-stimulators.

## Data Availability Statement

The datasets generated for this study are available on request to the corresponding authors.

## Ethics Statement

Ethical review and approval was not required for the study based on archived human biomaterials in accordance with national legislation and the code for usage of human remnant material (Code Goed Gebruik, Federation of Medical Scientific Societies in the Netherlands).

## Author Contributions

IJ participated in the research design, in performing laboratory experiments, interpreting the results, and writing the manuscript. MS contributed to the research design and set up of the experiments. MH participated in the histological analyses. AG participated in interpreting the results, histological analyses, and revising the manuscript. RP participated in the research design, interpreting the results, and revising the manuscript. All authors contributed to the article and approved the submitted version.

## Conflict of Interest

The authors declare that the research was conducted in the absence of any commercial or financial relationships that could be construed as a potential conflict of interest.

## Abbreviations

α -SMA: alpha-smooth muscle actin
CD45: cluster of differentiation 45
CK19: cytokeratin 19
DAPI: 4,6-diamidino-2-phenylindole
FISH: fluorescence *in situ* hybridization
H&E: hematoxylin and eosin
OLT: orthotopic liver transplantation
PBG: peribiliary gland
PBS: phosphate-buffered saline
PSC: primary sclerosing cholangitis
SSC: saline-sodium citrate buffer
vWF: von Willebrand factor.
