# Supplementary figures and images for: Evidence for Recipient-Derived Cells in Peribiliary Glands and Biliary Epithelium of the Large Donor Bile Ducts After Liver Transplantation
Source: Front Cell Dev Biol. 2020 Aug 5;8:693. doi: 10.3389/fcell.2020.00693 (PMC7419707; doi:10.3389/fcell.2020.00693)

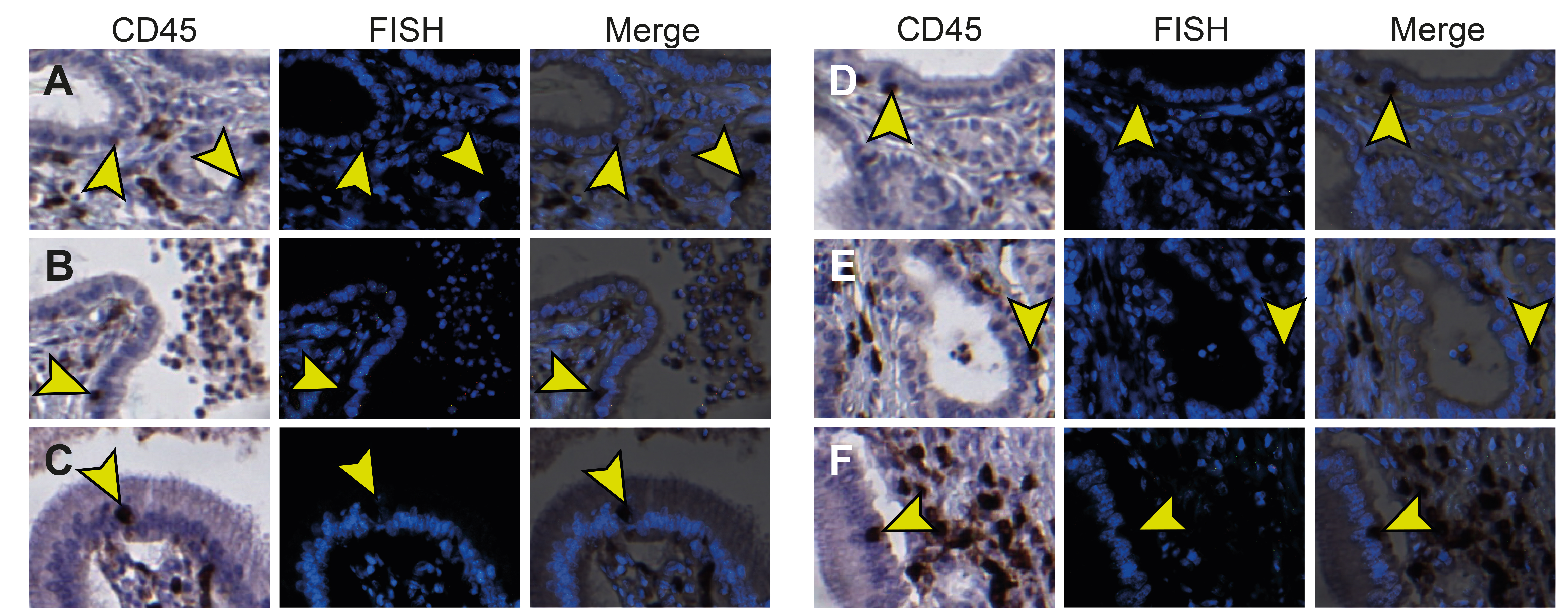

Supplement: Supplementary file 1 [file Image_1.JPEG]

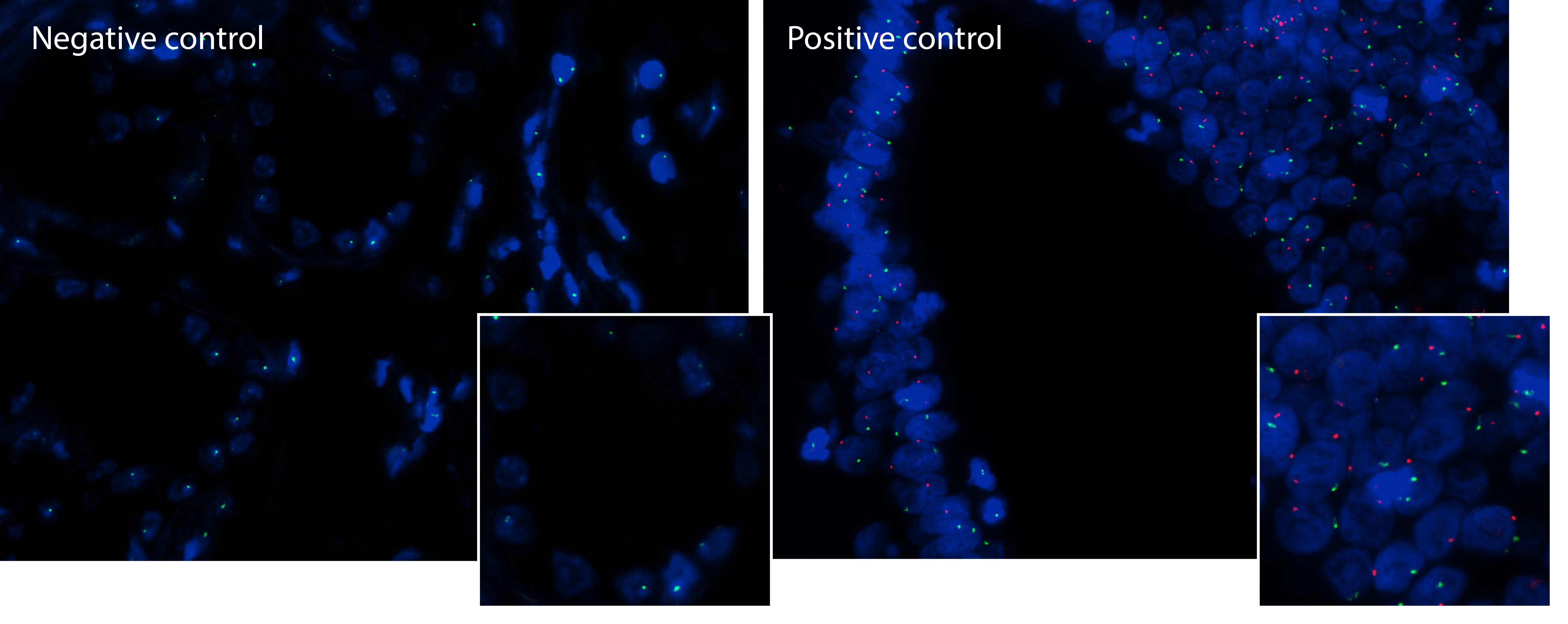

Supplement: Supplementary file 2 [file Image_2.JPEG]
